# Vitamin C plus hydrogel facilitates bone marrow stromal cell-mediated endometrium regeneration in rats

**DOI:** 10.1186/s13287-017-0718-8

**Published:** 2017-11-21

**Authors:** Huan Yang, Su Wu, Ran Feng, Junjiu Huang, Lixiang Liu, Feng Liu, Yuqing Chen

**Affiliations:** 1 Department of Obstetrics and Gynecology, the First Affiliated Hospital, Sun Yat-sen University, Guangzhou, 510080 China; 2 Center for Reproductive Medicine, the Seventh Affiliated Hospital, Sun Yat-sen University, Shenzhen, 518107 China; 3 Key Laboratory of Gene Engineering of the Ministry of Education, State Key Laboratory of Biocontrol, Institute of Healthy Aging Research, School of Life Sciences, Sun Yat-sen University, Guangzhou, 510006 China; 4 State Key Laboratory of Ophthalmology, Zhongshan Ophthalmic Center, Sun Yat-sen University, Guangzhou, 510275 China

**Keywords:** IUA endometrium, Pluronic F-127, BMSCs, Vitamin C

## Abstract

**Background:**

Intrauterine adhesion (IUA) is a common uterine cavity disease which can be caused by mechanical damage that may eventually lead to infertility and pregnancy abnormalities. Since the effect of therapeutic drugs appears disappointing, cell therapy has emerged as an alternative choice for endometrium regeneration. The aim of this study is to investigate whether the combination of hydrogel Pluronic F-127 (PF-127), Vitamin C (Vc), and a bone marrow stromal cell (BMSC) mixture could be a feasible strategy to improve the endometrial regeneration in a mechanical damage model of IUA in rats.

**Methods:**

Firstly, PF-127 cytotoxicity and the effect of Vc was tested in vitro using the Annexin V/propidium iodide (PI) apoptosis test, cell count kit (CCK) growth test, and enzyme-linked immunosorbent assay (ELISA). For the establishment of the rat IUA model, a 2-mm transverse incision in the uterus was prepared at the upper end, and 1.5- to 2.0-cm endometrial damage was scraped. Rats were randomly assigned to five groups to investigate the combined strategy on IUA uterine regeneration: a sham group, an IUA control group, an IUA BMSC encapsulated in PF-127 plus Vc group, an IUA BMSC plus Vc group, and an IUA PF-127 plus Vc group. A cell mixture was injected into the uterine horn while making the IUA model. Eight weeks after cell transplantation, the rats were sacrificed and the uterine was dissected for analysis. Endometrial thickness, gland number, fibrosis area, and the expression of marker proteins for endometrial membrane were examined by hematoxylin and eosin staining, Masson’s staining, and immunohistochemistry.

**Results:**

Vc promoted the survival and health of PF-127-encapsulated BMSCs in vitro. When this combination was transplanted in vivo, the endometrium showed better restoration as the endometrium membrane became thicker and had more glands and less fibrosis areas. The expression of cytokeratin, von Willebrand Factor (vWF), was also restored. The proinflammatory cytokine interleukin-1β (IL-1β) was significantly lower compared with the control group.

**Conclusions:**

Vc alleviates the cytotoxic effect of PF-127 and promotes cell survival and growth in rat BMSC encapsulation. Thus, a cell therapy strategy containing biomaterial scaffold, BMSCs and the modulatory factor Vc promotes the restoration of damaged IUA endometrium.

## Background

Intrauterine adhesion (IUA) is a common uterine cavity disease characterized by injury to the basal layer of the endometrium caused by mechanical injury or infection, leading to endometrial fibrosis, blockage of the uterine cavity, abnormal menstruation, and even infertility and pregnancy abnormalities [1–3]. Several therapeutic drugs have been tried in an attempt to improve the regeneration of the endometrium. However, most of these efforts were less effective and, in most situations, the results were controversial due to a diverse treatment schedule, a different population, and different measurements [4, 5].

Recent advances highlight cell therapy as an alternative choice for endometrium regeneration. Several sources of stem cells have been proposed for tissue regeneration, and bone marrow-derived mesenchymal stem cells are the most promising cell source of regeneration medicine due to their easy acquisition, self-renewal ability, multi-potential differentiation, and weak immunogenicity [6]. Bone marrow stromal cells (BMSCs) are a heterogeneous population of cells that reside in the bone marrow which also contains their main progenitors, mesenchymal stem cells. BMSCs can be distinguished from hematopoietic cells in bone marrow by plastic adherence, and can also be easily expanded in culture. BMSCs have immune modulatory properties and can induce angiogenesis and tissue repair, which makes them suitable for diverse applications in regeneration medicine [7].

Considering the tissue regenerative properties of BMSCs, we plan to transplant a high density of BMSCs into the damaged rat uterine cavity to achieve a better curative effect on endometrium regeneration. Therefore, an injectable material for BMSC encapsulation is urgently required for supporting the cells in the therapy. Pluronic F-127 (PF-127) is a synthetic Food and Drug Administration (FDA)-approved compound which has several therapeutic advantages, including low toxicity, biocompatibility, and thermo-reversibility [8–10]. A moderately concentrated solution of PF-127 in aqueous media (15–30%, w/w) is liquid at room temperature, but could form a hydrogel at physiological temperatures. Hence, PF-127 is widely used in drug delivery and in vivo tissue engineering [10–12]. Growing evidence has suggested that PF-127 might be a suitable scaffold for cell therapy using BMSCs [13]. However, PF-127 encapsulation is also reported to negatively affect cell survival and proliferation, which could be ameliorated by adding some membrane-stabilizing agents into the gel formulation [8].

Vitamin C (Vc), also known as l-ascorbic acid, has gained much public attention for its many functions contributing to the homeostasis of normal organs as well as tissue regeneration. Under Vc deprivation, human cells are unable to regenerate damaged tissue. At the same time, Vc has emerged as a key regulator of stem cells, influencing pluripotency, self-renewal, and differentiation [14]. Thus, in this study we proposed to test whether the hydrogel combination of PF-127-encapsulated BMSCs plus Vc is more effective and applicable for in vivo endometrial regeneration.

## Methods

### Animals

Eight-week-old Sprague-Dawley rats (female) weighting 200–250 g were used in all experiments. All animal experiments were performed according to protocols approved by the Committee on Animal Care at the School of Life Sciences, Sun Yat-Sen University, China.

### BMSC isolation from female rats

BMSCs were isolated according to a modified protocol from a previous report [15]. Briefly, 8-week-old female Sprague-Dawley rats were sacrificed by intraperitoneal (i.p.) injection of 50% chloral hydrate solution (Guangzhou, China), and then sterilized in 75% ethanol (Guangzhou, China). The femurs and tibiae were collected and BMSCs were flushed out using Dulbecco’s modified Eagle’s medium (DMEM)/F12 (Gibco, Grand Island, NY). The suspended cells were centrifuged at 200 g for 5 min, and then seeded in DMEM/F12 media with 10% fetal bovine serum (FBS) and 1% penicillin/streptomycin (Gibco, Grand Island, NY) at 37 °C in an incubator with 5% CO_2_. The non-adherent cells were removed 24 h later and the culture medium was changed every 3 days. The BMSCs cultured to the third passage were used for the in-vitro test and in-vivo transplantation.

### PF-127 preparation and cell encapsulation

PF-127 was dissolved in DMEM/F12 at 4 °C overnight to prepare a 20% (w/v) solution. The solution was then filtered through a 0.45-μm filter (Millipore, Billerica, MA) and finally kept at 4 °C for use. For the in-vitro experiment, BMSCs were centrifuged and resuspended in sterile PF-127 solution with 0/50/100 μM Vc (A4403, Sigma, St. Louis, MO) on ice; 200 μL of cell-hydrogel mixture was added to each well, and the plate then was kept in a 37 °C incubator (5% CO_2_) for 5 min to boost gel formation. A further 0.5 mL culture medium was added over the gel and the plate was transferred back into the incubator. For the in-vivo experiment, 8-week-old female rats were randomly divided into five groups: sham (no surgery), control (IUA without injection of BMSCs or PF-127/Vc), BMSC encapsulation (IUA and injection of BMSCs capsulated with PF-127/Vc), BMSC plus Vc (IUA and injection of BMSCs only with Vc), and PF-127 plus Vc (IUA and injection of only F-127/Vc). Cells (8 × 10^5^) were injected in a total volume of 200 μL.

### Fluorescence-activated cell sorting (FACS) analysis

The third passage of rat BMSCs were digested with 0.05% trypsin-EDTA (Gibco, Grand Island, NY) and 10^5^ cells were resuspended in 100 μL DMEM/F12. Resuspended cells were incubated with 5 μL phycoerythrin (PE)-conjugated anti-rat CD34 (12-0349, ebioscience, Waltham, MA), anti-rat CD45 (12-0461, ebioscience, Waltham, MA), anti-rat CD29 (551401, BD Pharmingen™, Franklin Lake, NJ), and anti-rat CD90 (555005, BD Pharmingen™, Franklin Lake, NJ) in the dark on ice for 30 min. Cytometric analysis was performed using a flow cytometer (CytoFLEX, Beckman Coulter, Indianapolis, IN).

### Osteogenesis and adipogenesis induction of rat BMSCs

Cells were characterized for their capability to differentiate into osteoblasts or adipocytes using commercial osteogenesis and adiopogenesis induction kits, following the manufacturer’s instructions (RASMX-90021 and RASMX-90031, Cyagen, Santa Clara, CA). Osteoblasts were stained with Alizarin Red S and adipocytes were stained with Oil Red O.

### Cell counting kit (CCK) cell proliferation assay

BMSC proliferation was assessed by the TransDetect™ cell counting kit (TransGen Biotech, Beijing, China) following the manufactory’s instructions. Briefly, the BMSCs were seeded at a final density of 1 × 10^5^/mL under different encapsulation conditions. Cell growth at day 3 and 7 after seeding were tested by adding 10% CCK to each well and incubating for 2 h before measuring the absorbance at 450 nM using a microplate reader (VICTOR™ X5, PerkinElmer, Waltham, MA).

### Cell apoptosis assay

The TransDetect Annexin V-FITC/PI Apoptosis Detection Kit (TransGen Biotech, Beijing, China) was used to evaluate cellular apoptosis according to the manufacturer’s instructions. Briefly, BMSCs were seeded at a final density of 1 × 10^5^/mL under different encapsulation conditions for 3 days and 7 days; the cells were then trypsinized and suspended in binding buffer labeled with 5 μL Annexin V-FITC and 5 μL propidium iodide (PI) for 15 min in the dark on ice. Finally, 300 μL binding buffer was added to each sample and the cells were evaluated by a flow cytometer. Positive control was carried out using 100 μM H_2_O_2_ treatment for 15 min.

### Enzyme-linked immunosorbent assay (ELISA)

BMSCs (10^5^/mL) in 24-well plates were seeded under different encapsulation conditions, and the culture supernatants were harvested 3 days later. Rat hepatocyte growth factor (HGF), insulin-like growth factor (IGF), epidermal growth factor (EGF), interleukin (IL)-10, IL-6, and tumor necrosis factor (TNF)-α were measured using commercial ELISA Kits (Boster, Wuhan, China) according to the manufacturer’s instructions.

### Rat IUA model and BMSC transplantation

A rat uterine cavity adhesion model was established using the mechanical damage method. Briefly, rats were anesthetized with 10% chloral hydrate (300 mg/kg) and the abdominal cavity was opened to expose the uterus. A 2-mm transverse incision in the left uterus was prepared at the upper end, and a 1.5- to 2.0-cm rough-feeling endometrial damage was generated by a scraping spoon. In the BMSC transplantation test, 200 μL PF-127-encapsulated BMSCs with Vc or other controls was injected into the uterine horn while establishing the IUA model. After the surgery, the rat abdomen was sutured and the rat was allowed to recover for 8 weeks followed by further examination. The right-sided uterus without damage was considered as the sham control.

### Histological analysis

Eight weeks after surgery, the rats were sacrificed and the tissues underwent standard paraffin embedding, section cutting, and hematoxylin and eosin (H&E) staining. The morphological changes were observed under the light microscope. Five fields in each image were selected for counting. Image Pro-Plus 6.0 (IPP 6.0) was applied to analyze the thickness of the endometrium, the total number of endometrial glands, and the area of interstitial fibrosis of the endometrium. Endometrial fibrosis was revealed by Masson’s trichrome staining. Briefly, 8 weeks after surgery, the rats were sacrificed and the tissues underwent standard paraffin embedding, and 4-μm serial sections were prepared. Sections were immune-labeled with anti-IL-1β antibody (1:150, rabbit, Bioss, Beijing, China), anti-Pan Cytokeratin antibody (1:50, mouse, Boster, Wuhan, China), and anti-von Willebrand factor (vWF) antibody (1:70, mouse, Boster, Wuhan, China). Percentages of the positive staining area were quantified using the Image-Pro Plus software (Media Cybernetics, Rockville, MD).

### Statistical analyses

All the data are presented as mean ± standard deviation (SD) and analyzed by GraphPad Prism (La Jolla, CA). Multiple group comparisons were determined by either two-way analysis of variance (ANOVA) or one-way ANOVA followed by Bonferroni’s post-hoc test. Histological measurement difference was determined by Student’s *t* test. *P* < 0.05 was considered statistically significant.

## Results

### Isolation of BMSCs from rats

**Fig. 1:**
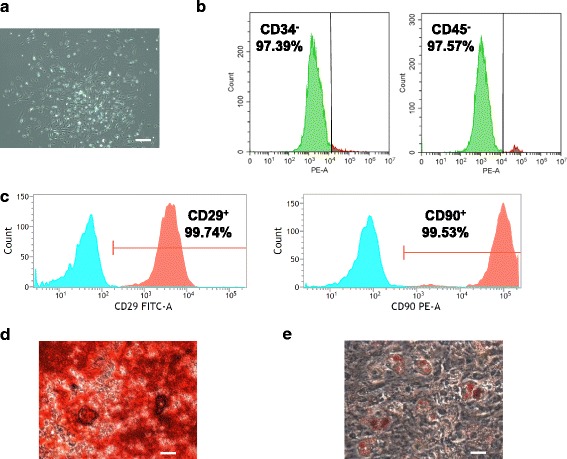
Preparation of rat bone marrow stromal cells. **a** The typical morphology of rat bone marrow stromal cells at the second passage under light microscopy. *Scale bar* = 10 μm. **b** Flow cytometry analysis of hematopoietic markers (CD34/CD45) from the isolated bone marrow stromal cells. The majority of the isolated cells were CD34/CD45 negative. **c** Flow cytometry analysis of mesenchymal markers (CD29/CD90) from the isolated bone marrow stromal cells. The majority of the isolated cells were CD29/CD90 positive. **d** Osteogenesis of isolated rat BMSCs. Osteoblast differentiation is indicated by Alizarin Red S staining. *Scale bar* = 2 μm. **e** Adipogenesis of isolated rat BMSCs. Adipocyte differentiation is indicated by Oil Red O staining. *Scale bar* = 2 μm

Rat BMSCs were derived from tibiae and femurs of 8-week-old Sprague-Dawley female rats by bone marrow aspiration following published methods [16]. The cultured BMSCs were adherent to plastic dishes. A typical phenotype of the rat bone marrow colony is shown in Fig. 1a. As mesenchymal stromal cells express cluster of differentiation 29 (CD29) and CD90, but lack expression of CD34 and CD45 on the cell surface [17, 18], we sought to test the expression of these markers in our cultured BMSCs. FACS analysis showed that the majority of the cultured cells were negative for hematopoietic markers after culturing for 7 days (97.39% for CD34^–^ and 97.57% for CD45^–^), but highly positive for CD29 (99.74%) and CD90 (99.53%) expression (Fig. 1b and c). Moreover, our cultured BMSCs could differentiate into osteoblasts and adipocytes in vitro (Fig. 1d and e), indicating that the cultured cell population were multi-potent mesenchymal stromal cells. Thus, these cells were used for further ex vivo experiments and in vivo transplantation.

### Vitamin C promotes rat BMSC survival in PF-127 hydrogel encapsulation

**Fig. 2:**
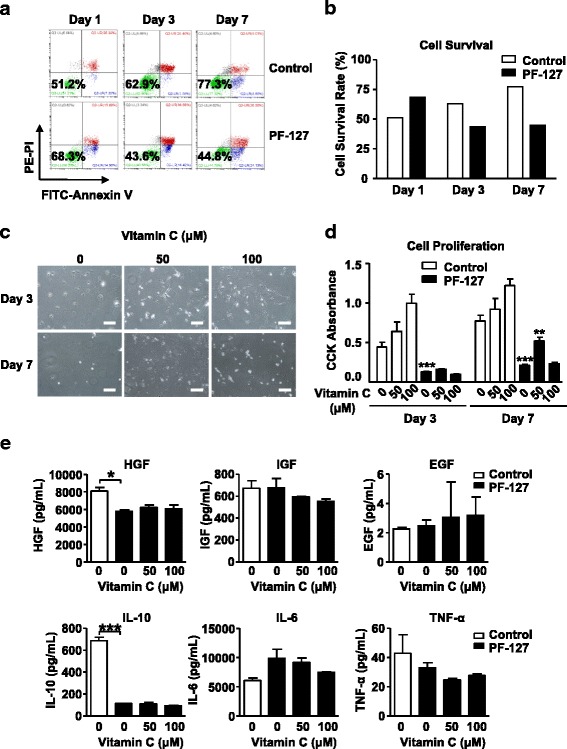
Vitamin C promotes rat bone marrow stromal cell survival in vitro. **a** Flow cytometry analysis of cell apoptosis on days 1, 3, and 7 after seeding. *Upper row*: BMSCs in normal culture medium; *lower row*: BMSC encapsulated in PF-127 hydrogel (20%, w/v). **b** Quantification of BMSC apoptosis flow cytometry analysis, with and without PF-127. **c** The morphology of rat bone marrow stromal cells cultured in PF-127 supplemented with indicated concentrations of vitamin C for 3 days and 7 days. *Scale bar* = 10 μm. **d** Cell count kit (*CCK*) assay showing the cell growth of BMSCs in PF-127 supplemented with 0 μM, 50 μM, and 100 μM vitamin C at day 3 and day 7. Two-way ANOVA, followed by Bonferroni post-hoc tests; **P < 0.01, ***P < 0.001. **e** ELISA results show the concentration of major growth factors and cytokines produced by rat bone marrow stromal cell at day 3 after seeding. Data are presented as mean ± SD. Student’s *t* test, *n* = 3; *P < 0.05, ***P < 0.001. *EGF* epidermal growth factor, *HGF* hepatocyte growth factor, *IGF* insulin-like growth factor, *IL* interleukin, *TNF* tumor necrosis factor

We first tested whether PF-127 encapsulation could affect rat BMSC growth. BMSCs were seeded at a density of 1 × 10^5^/mL in 20% (w/v) PF-127 hydrogel. Cell apoptosis was examined by FACS using Annexin V/PI double staining, and the cell survival rate was calculated based on the FACS data. In the early stages, there was a short period of survival enhancement (68.3% versus 51.2%) after PF-127 encapsulation on the first day after seeding (Fig. 2a and b; day 1). However, after 3 days of culture, the majority of stromal cells encapsulated in PF-127 started undergoing apoptosis (43.6% versus 62.9%) (Fig. 2a and b; day 3). After 7 days of culture, the majority of cells encapsulated in PF-127 with no addition of Vc underwent apoptosis (44.8% versus 77.3%) and detached due to PF-127 cytotoxicity (Fig. 2a and b; day 7). However, Vc significantly improved in-vitro cell attachment at 7 days, while adding 50 μM Vc showed a better effect than 100 μM (Fig. 2c). The CCK test also showed a similar cytotoxic effect of PF-127 (Fig. 2d; day 3 and day 7, PF-127 versus control), and that cells encapsulated in PF-127 supplemented with 50 μM Vc proliferated faster over a longer term of 7 days of culture (Fig. 2d; 50 μM versus 100 μM), indicating that 50 μM Vc improved cell viability. Moreover, PF-127 encapsulation obviously changed the cytokine profile, looking at HGF and IL-10 (Fig. 2e). More importantly, Vc did not affect the production of most growth factors and cytokines from rat BMSCs (Fig. 2e). Taken together, Vc promoted PF-127-encapsulated BMSCs viability in vitro, and we chose 50 μM Vc for the following experiments.

### Establishment of rat IUA model

**Fig. 3:**
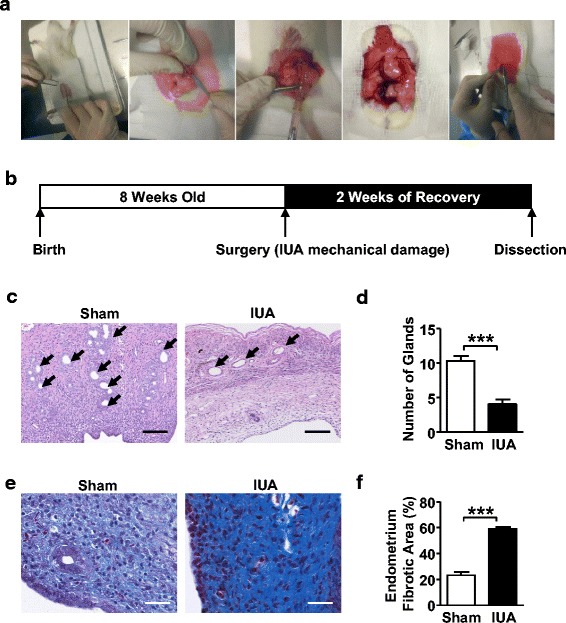
Establishment of a rat IUA model. **a** General surgery procedures. Rats were anesthetized by intraperitoneal injection of 10% chloral hydrate (300 mg/kg), and a vertical incision was made in the lower abdomen to expose the uterine horns. The uterine wall (approximately 0.2 cm) was excised 0.5 cm from the left uterine horn, and then mechanical damage of the upper 1.5–2.0 cm of the endometrium was generated with spoon scratching until the inner membrane had a rough feeling. Finally, the rectus fascia and skin were sealed with a 4-0 nylon suture. **b** Schematic representation of the in-vivo IUA model set up. Three 8-week-old Sprague-Dawley rats were assigned as the intrauterine adhesion (*IUA*) group with mechanical damage in the left uterin cavity; the other side of the uterine cavity was assigned as the control sham group (*n* = 3). Two weeks after surgery, the rats were sacrificed and the uterus was dissected for histological examination. **c** H&E staining of the IUA endometrium compared with the sham control group. *Scale bar* = 200 μm. **d** Statistical results of endometrial gland number in sham control and IUA rat uterine cavity. Data are presented as mean ± SD. Student’s *t* test, *n* = 3; ***P < 0.001. **e** Masson staining of the IUA endometrium compared with the sham control group. **f** Statistical results of the percentage of endometrial fibrotic area in rat uterine cavity. Data are presented as mean ± SD. Student’s *t* test, *n* = 3; ***P < 0.001

In order to test the in-vivo effect of a BMSC/PF-127/Vc combination strategy for tissue regeneration, we first carried out rat uterine mechanical damage surgery (Fig. 3a). Eight-week-old female Sprague-Dawley rats were mechanically damaged on the left uterine endometrial membrane to generate the IUA model, and the right side uterus without damage was considered as the sham control. After 2 weeks of recovery, rats were sacrificed and dissected for examination (Fig. 3b). In the sham control rats, the glands were uniformly distributed on the surface of the uterine endometrial cavity, and collagen fibers were barely observed by Masson staining. Histologic evaluation of the IUA uterus revealed that there was a significant decrease in the number of glands in the IUA uterine cavities compared with the sham counterparts (Fig. 3c and d). In contrast, the fibrotic area in the IUA uterus was significantly increased by Masson staining (Fig. 3e and f). Together, these results showed that mechanical damage leads to a severe intrauterine abnormality, suggesting a successful establishment of a rat IUA model.

### PF127-embeded BMSCs promote the regeneration of uterine endometrium in the rat IUA model

**Fig. 4:**
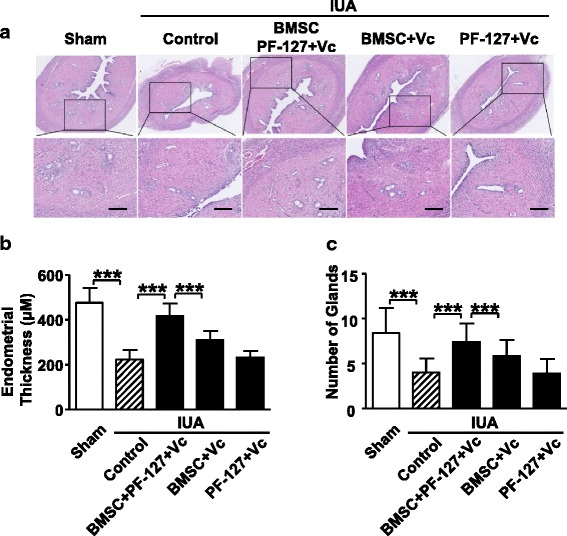
PF-127-embedded bone marrow stromal cells (*BMSCs*) restored endometrial thickness and gland number in the rat intrauterine adhesion (*IUA*) model. **a** H&E staining of treated IUA endometrium compared with control groups. *Scale bar* = 200 μm. **b** Statistical resultss of endometrial thickness in rat IUA groups with different treatments. Data are presented as mean ± SD. Student’s *t* test analysis, *n* = 6; ***P < 0.001. **c** Statistical results of endometrial gland number in control and different treated IUA rat uterine cavities. Data are presented as mean ± SD. Student’s *t* test analysis, *n* = 6; ***P < 0.001. *Vc* vitamin C

In order to test whether PF-127 with Vc encapsulation would improve IUA recovery in vivo, the mechanically injured rats were transplanted with BMSCs (3 × 10^6^/mL) from 8-week-old female donors encapsulated in PF-127 supplemented with 50 μM Vc (BMSC/PF-127/Vc) in the left uterine cavity immediately after IUA establishment. In addition to the IUA control group, injured rats were also injected with BMSCs plus 50 μM Vc (BMSC/Vc) or only with PF-127 plus 50 μM Vc (PF-127/Vc) as a strict control. Eight weeks after the transplantation, rats were sacrificed for histopathological analysis. H&E staining showed that the IUA rat uterine cavity in the BMSC/PF-127/Vc group recovered best (Fig. 4a), as the restored endometrial thickness (Fig. 4b) and the number of glands (Fig. 4c) was very similar to those in the sham group. On the contrary, the endometrial thickness and number of glands in the BMSC/Vc group were partially rescued, but was significantly less satisfactory without the hydrogel encapsulation. For the PF-127/Vc group without BMSCs, there was no rescue effect at all (Fig. 4).

**Fig. 5:**
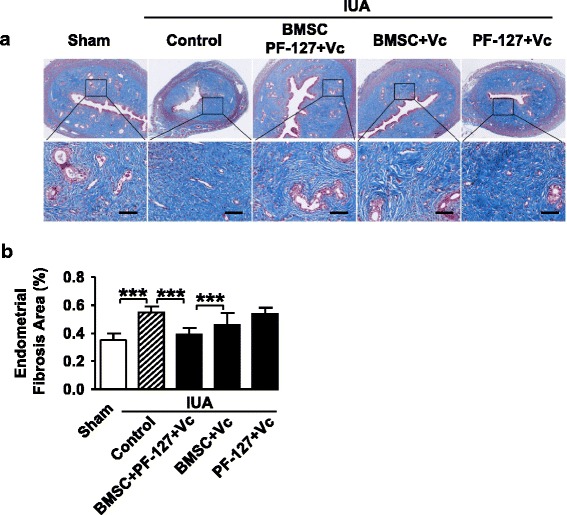
PF-127-embedded bone marrow stromal cells (*BMSCs*) restored the endometrial fibrosis area in the rat intrauterine adhesion (*IUA*) model. **a** Masson staining of treated IUA endometrium compared with the control group. *Scale bar* = 200 μm. **b** Statistical results of the endometrial fibrosis area in rat IUA groups with different treatments. Data are presented as mean ± SD. Student’s *t* test, *n* = 12 in sham/control group, *n* = 8 in other IUA groups; ***P < 0.001. *Vc* vitamin C

**Fig. 6:**
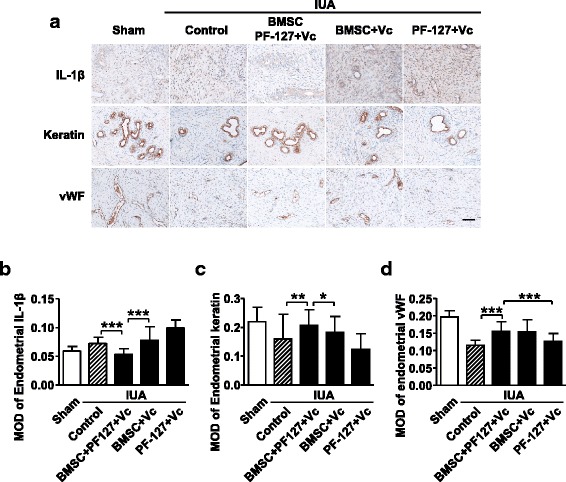
PF-127-embedded bone marrow stromal cells (*BMSCs*) promote uterine endometrium regeneration in the rat intrauterine adhesion (*IUA*) model. **a** IL-1β/keratin/vWF staining of the IUA endometrium. *Scale bar* = 200 μm. Statistical results of **b** endometrial interleukin-1beta (*IL-1β*), **c** endometrial keratin, and **d** endometrial von Willebrand factor (*vWF*) production in rat IUA groups with different treatments. Data are presented as mean ± SD. Student’s *t* test, *n* = 12 in sham/control group, *n* = 8 in other IUA groups; ***P < 0.001. *Vc* vitamin C

Masson staining also indicated that BMSC/PF-127/Vc transplantation in the rat uterine cavity significantly reduced the fibrotic area of the endometrial stromal, but other IUA groups remained with high levels of endometrial fibrosis (Fig. 5). Immunohistochemistry showed less endometrial IL-1β but more keratin and endometrial vWF production when restored by BMSC/PF-127/Vc transplantation, while the other transplanted groups did not show any similar effects (Fig. 6).

## Discussion

It is widely accepted that multi-potential bone marrow mesenchymal stromal cells could be used for tissue regeneration [19]. The aim of this research was to restore mechanically damaged rat uterine endometrium using bone marrow stromal cells (BMSCs). The advantage of using BMSCs relies firstly on it being more convenient to obtain them by simple cell adhesion of bone marrow after excluding blood cells. Secondly, there is an overwhelming number of BMSCs to reach the transplantation density. Thirdly, the BMSCs are a mixture of several cell types, including progenitor stem cells, fibroblasts, and immune cells, that may offer better immune-regulatory ability in the tissue regeneration process.

Thermo-reversible gelation of PF-127 makes it an ideal carrier for cell encapsulation to help cell attachment as a scaffold, but it is also true that there is a decrease in cell viability as the gel concentration increases [9]. We found significant cell apoptosis and growth inhibition in 20% (w/v) PF-127, a concentration widely used for cell encapsulation. Therefore, we tried to find a method to reduce the negative effect of PF-127 on cell survival. Several agents, such as glucose, glycerol, and hydrocortisone, have been reported to maintain cell viability over time [8]. In this study, we proposed vitamin C (Vc) as a new effective membrane stabilizing agent. We found Vc significantly improved rat BMSC viability in vitro after 7 days of culture. This effect is very likely due to the fact that Vc could influence the extracellular matrix (ECM) and collagen homeostasis [14]. It is also known that Vc has an anti-inflammatory function and so it could be co-applied as an adjuvant to regulate the immune response [20]. Similarly in our ELISA results, there was a decreased trend of IL-6 and TNF-α secretion after adding Vc during the cell encapsulation (Fig. 2e).

Vc is a strong reducing substance and cooperates to maintain intracellular reactive oxygen species (ROS) levels, which not only regulates several signaling pathways involved in pluripotency [21], but also contributes to slowing down the cellular aging process [22]. In our CCK cell growth and apoptosis analysis, BMSCs encapsulated by PF-127 plus Vc showed significantly improved cell survival rate (Fig. 2b and d). Reduced ROS pressure by Vc could be one of the possible reasons for the long-term health of BMSCs in encapsulation. We noticed that a low concentration (50 μM) of Vc worked better than a high concentration (100 μM), and this may due to 100 μM of Vc apparently changing the hydrogel encapsulation pH value which possibly affected cell proliferation. In recent years, a series of reports unveiled Vc as a regulator of stem cell pluripotency, self-renewal, and the generation of induced pluripotent stem cells (iPSCs) [23, 24]. Taken together, Vc could benefit multiple cell types in the stromal cell mixture, which finally facilitates overall tissue regeneration.

The immune system plays a central role in tissue repair and regeneration, which determines the speed and the outcome of the healing process. It is well recognized that the innate immune response, such as danger signals, neutrophils, and macrophages, as well as the adaptive immune response can modulate tissue healing [25]. Implanted biomaterials could have a significant intrinsic effect on the immune system. As shown in Fig. 2b and e, PF-127 significantly affected cell viability and growth factor/cytokine production. After adding Vc, it not only partially rescued the overproduced inflammatory cytokines (IL-6 and TNF-α), but also promoted 7-day cell survival. IL-10 is a main anti-inflammatory cytokine secreted by mesenchymal cells [26]. PF-127 encapsulation may enhance the inflammatory response as it significantly reduces IL-10 expression of BMSCs which cannot be rescued by adding Vc. This result suggests that Vc enhances PF-127-encapsulated BMSC survival via an IL-10- independent manner. Although the detailed mechanisms as to how these immune conditions affect endometrial regeneration are still unclear, here we provide an alternative therapy strategy combining biomaterial scaffold, stromal cell mixture, and immune regulation to better promote the restoration of damaged tissues and organs.

Because of its high solubility in water, Vc is easy to wash away after cell implantation. Therefore, hydrogel encapsulation is good for preserving Vc and BMSCs in the wound area. In summary, we found that Vc plus PF-127-encapsulated BMSCs is applicable in endometrial regeneration. Together, our study provides an easily accessible cell source as well as a feasible supporting method for cell growth and delivery in endometrium regeneration, which may also have pre-clinical implications. For clinic use, BMSCs from either the patient themself or other origins, such as human umbilical mesenchymal stromal cells, may be ideal cell sources for endometrium regeneration.

## Conclusions

In this study, we found Vc significantly promotes PF-127-encapsulated BMSC survival and growth in vitro, functioning as a potent modulator to alleviate the cytotoxic effect of hydrogel PF-127. In a rat IUA model, the combination strategy including biomaterial scaffold (PF-127), BMSCs, and Vc improves the restoration of damaged IUA endometrium in vivo. In conclusion, Vc plus PF-127 hydrogel could be a promising approach for supporting BMSCs in cell therapy.

## Abbreviations

ANOVA: Analysis of variance
BMSC: Bone marrow stromal cell
CCK: Cell count kit
CD: Cluster of differentiation
DMEM: Dulbecco’s modified Eagle’s medium
ECM: Extracellular matrix
EGF: Epidermal growth factor
ELISA: Enzyme-linked immunosorbent assay
FACS: Fluorescence-activated cell sorting
FBS: Fetal bovine serum
H&E: Hematoxylin and eosin
HGF: Hepatocyte growth factor
IGF: Insulin-like growth factor
IL: Interleukin
iPSC: Induced pluripotent stem cell
IUA: Intrauterine adhesion
PE: Phycoerythrin
PI: Propidium iodide
ROS: Reactive oxygen species
SD: Standard deviation
TNF: Tumor necrosis factor
Vc: Vitamin C
vWF: Von Willebrand Factor

## References

[1] Yu D (2008). Asherman syndrome—one century later. Fertil Steril.

[2] Zupi E, Centini G, Lazzeri L (2015). Asherman syndrome: an unsolved clinical definition and management. Fertil Steril.

[3] March CM (2011). Management of Asherman's syndrome. Reprod Biomed Online.

[4] Zhao M (2010). Treatment with low-dose aspirin increased the level LIF and integrin beta3 expression in mice during the implantation window. Placenta.

[5] Sher G, Fisch JD (2002). Effect of vaginal sildenafil on the outcome of in vitro fertilization (IVF) after multiple IVF failures attributed to poor endometrial development. Fertil Steril.

[6] Nelson TJ (2009). Stem cell platforms for regenerative medicine. Clin Transl Sci.

[7] Stroncek DF (2014). Establishing a bone marrow stromal cell transplant program at the National Institutes of Health Clinical Center. Tissue Eng Part B Rev.

[8] Khattak SF, Bhatia SR, Roberts SC (2005). Pluronic F127 as a cell encapsulation material: utilization of membrane-stabilizing agents. Tissue Eng.

[9] Matthew JE (2002). Effect of mammalian cell culture medium on the gelation properties of Pluronic F127. Biomaterials.

[10] Ruszymah BH (2005). Formation of in vivo tissue engineered human hyaline cartilage in the shape of a trachea with internal support. Int J Pediatr Otorhinolaryngol.

[11] Escobar-Chavez JJ (2006). Applications of thermo-reversible pluronic F-127 gels in pharmaceutical formulations. J Pharm Pharm Sci.

[12] Cortiella J (2006). Tissue-engineered lung: an in vivo and in vitro comparison of polyglycolic acid and pluronic F-127 hydrogel/somatic lung progenitor cell constructs to support tissue growth. Tissue Eng.

[13] Vashi AV (2008). Adipose differentiation of bone marrow-derived mesenchymal stem cells using Pluronic F-127 hydrogel in vitro. Biomaterials.

[14] D'Aniello C (2017). Vitamin C in stem cell biology: impact on extracellular matrix homeostasis and epigenetics. Stem Cells Int..

[15] Jing Z (2014). Rat bone marrow mesenchymal stem cells improve regeneration of thin endometrium in rat. Fertil Steril.

[16] Munoz-Elias G, Woodbury D, Black IB (2003). Marrow stromal cells, mitosis, and neuronal differentiation: stem cell and precursor functions. Stem Cells.

[17] Harting M (2008). Immunophenotype characterization of rat mesenchymal stromal cells. Cytotherapy.

[18] Dominici M (2006). Minimal criteria for defining multipotent mesenchymal stromal cells. The International Society for Cellular Therapy position statement. Cytotherapy.

[19] Wang J (2016). Application of bone marrow-derived mesenchymal stem cells in the treatment of intrauterine adhesions in rats. Cell Physiol Biochem.

[20] Sorice A (2014). Ascorbic acid: its role in immune system and chronic inflammation diseases. Mini Rev Med Chem.

[21] Frei B, England L, Ames BN (1989). Ascorbate is an outstanding antioxidant in human blood plasma. Proc Natl Acad Sci U S A.

[22] Chandrasekaran A, Idelchik MD, Melendez JA (2017). Redox control of senescence and age-related disease. Redox Biol..

[23] Esteban MA (2010). Vitamin C enhances the generation of mouse and human induced pluripotent stem cells. Cell Stem Cell.

[24] D'Aniello C (2017). Vitamin C and l-proline antagonistic effects capture alternative states in the pluripotency continuum. Stem Cell Reports.

[25] Julier Z (2017). Promoting tissue regeneration by modulating the immune system. Acta Biomater..

[26] Najar M (2016). Mesenchymal stromal cells and immunomodulation: a gathering of regulatory immune cells. Cytotherapy.

